# WHIRLY1 functions in the nucleus to regulate barley leaf development and associated metabolite profiles

**DOI:** 10.1042/BCJ20210810

**Published:** 2022-03-04

**Authors:** Barbara Karpinska, Nurhayati Razak, Euan K. James, Jenny A. Morris, Susan R. Verrall, Peter E. Hedley, Robert D. Hancock, Christine H. Foyer

**Affiliations:** 1 School of Biosciences, College of Life and Environmental Sciences, University of Birmingham, Edgbaston, Birmingham B15 2TT, U.K.; 2 Environmental Sciences, The James Hutton Institute, Invergowrie, Dundee DD2 5DA, U.K.; 3 Cell and Molecular Sciences, The James Hutton Institute, Invergowrie, Dundee DD2 5DA, U.K.

**Keywords:** cell nucleus, chloroplast, development, photosynthesis

## Abstract

The WHIRLY (WHY) DNA/RNA binding proteins fulfil multiple but poorly characterised functions in leaf development. Here, we show that WHY1 transcript levels were highest in the bases of 7-day old barley leaves. Immunogold labelling revealed that the WHY1 protein was more abundant in the nuclei than the proplastids of the leaf bases. To identify transcripts associated with leaf development we conducted hierarchical clustering of differentially abundant transcripts along the developmental gradient of wild-type leaves. Similarly, metabolite profiling was employed to identify metabolites exhibiting a developmental gradient. A comparative analysis of transcripts and metabolites in barley lines (W1–1 and W1–7) lacking WHY1, which show delayed greening compared with the wild type revealed that the transcript profile of leaf development was largely unchanged in W1–1 and W1–7 leaves. However, there were differences in levels of several transcripts encoding transcription factors associated with chloroplast development. These include a barley homologue of the Arabidopsis GATA transcription factor that regulates stomatal development, greening and chloroplast development, NAC1; two transcripts with similarity to Arabidopsis GLK1 and two transcripts encoding ARF transcriptions factors with functions in leaf morphogenesis and development. Chloroplast proteins were less abundant in the W1–1 and W1–7 leaves than the wild type. The levels of tricarboxylic acid cycle metabolites and GABA were significantly lower in WHY1 knockdown leaves than the wild type. This study provides evidence that WHY1 is localised in the nuclei of leaf bases, contributing the regulation of nuclear-encoded transcripts that regulate chloroplast development.

## Background

Cellular compartmentalisation is essential for the regulation of metabolism and gene expression [1]. Reciprocal communication between the mitochondria, chloroplasts and nuclei is not only vital for the efficient functions of these compartments, but it also ensures the rapid adjustment of their protein content and composition to changing environmental conditions. Mitochondria- and plastid-derived retrograde signals are therefore important components in the regulation of nuclear gene expression [2–5]. In plastids, transcription is under the control of two types of RNA polymerases, a unique eubacterial-type plastid-encoded polymerase (PEP) and phage-type nucleus-encoded polymerases (NEPs). These RNA-polymerases specifically regulate the transcription of different subsets of genes but can also co-regulate a portion of the plastidial genes. The formation of chloroplasts from proplastids requires the establishment of the PEP complex. The PEP complex is composed of a catalytic core comprising plastid-encoded proteins (*rpoA*, *rpoB*, *rpoC1* and *rpoC2*) and additional polymerase-associated proteins (PAP) including other nuclear-encoded polymerase-associated proteins and sigma factors (SIGs). The latter are required by PEP for promoter recognition [6]. PEP status/activity provides positive retrograde signals from the chloroplasts that convey essential information to the nucleus to promote *PhANG* (photosynthesis-associated nuclear gene) expression.

Mitochondria to nucleus signalling, which involves two key transcription factors; *ANAC013* and *ANAC017*, is also linked to plastid to nucleus signalling [7]. The *ANAC013* and *ANAC017* transcription factors are released from the endoplasmic reticulum upon perception of appropriate signals and translocated to the nucleus, where they activate the expression of a specific set of genes called mitochondrial dysfunction stimulon (MDS) genes that include the alternative oxidases, *SOT12* and *ANAC013* [8,9]. The enhanced expression of *ANAC013* provides positive feedback regulation of the signalling pathway. The nuclear-localised RADICAL-INDUCED CELL DEATH1 (RCD1) protein suppresses ANAC013 and ANAC017 functions [7]. In addition, SOT12 belongs to the group of MDs genes that overlap with the genes induced by the SAL1, 3′-phosphoadenosine 5′-phosphate (PAP) chloroplast retrograde signalling pathway [10].

The WHY family of proteins, which are specific to the plant kingdom [11] have a putative KGKAAL DNA binding domain that allows binding to ssDNA molecules of differing nucleotide sequence [12] which may allow them to function as PAPs allowing the possibility of a functional interaction between these proteins [2]. All plants have two *WHY* genes (*WHY1* and *WHY2*). *WHY1* encodes a protein that is located in chloroplasts and nuclei while *WHY2* encodes a mitochondria-targeted protein [13]. WHY1 protein interacts with thylakoid membrane proteins and with the chloroplast nucleoids [14,15]. Unlike many other species, Arabidopsis has a third WHY gene, *AtWHY3* that is targeted to plastids [16]. However, the intracellular localisation of the WHY proteins appears to be flexible and determined by developmental and environmental signals. For example, the WHY2 protein that is primarily associated with mitochondrial nucleoids, was found in mitochondria, chloroplasts and nuclei during leaf senescence [17]. Moreover, it appears that WHY3 can compensate for WHY2 in the Arabidopsis *why 2-1* mutant because WHY3 can be targeted to both chloroplasts and mitochondria [18]. The expression of WHY2 in Arabidopsis decreased the expression of genes encoded by the chondriome [19]. Similarly, the expression of the tomato SlWHY2 in transgenic tobacco plants led to mitochondrial gene transcription and stabilisation of mitochondrial functions [20].

Barley leaves deficient in the WHY1 protein have higher levels of chlorophyll than the wild type with an enhanced abundance of plastome-encoded transcripts [21,22]. In contrast, the leaves of the Arabidopsis *why1* mutant and *why1why3* double mutants are phenotypically similar to the wild type. However, a *why1why3polIb-1* triple mutant defective in WHY1, WHY3, and the DNA polymerase 1B (Pol1B) exhibited a severe yellow-variegated phenotype [23]. WHY1, WHY3 and RECA1 (a plastid localised DNA recombination-family protein involved in DNA repair) are associated with the chloroplast RNase H1 AtRNH1C protein and work together to maintain chloroplast genome integrity [24]. Maize transposon insertion lines in *WHY1* (Zmwhy1-1) have equivalent amounts of chloroplast DNA (cpDNA) to the wild type but are deficient in plastid ribosomes resulting in an albino phenotype [25].

We have previously characterised the phenotypes, and metabolite and transcriptome profiles of three RNAi-knockdown barley lines (W1–1, W1–7 and W1–9) that have very low levels of *HvWHY1* expression [21]. The formation of chloroplast ribosomes and the establishment of photosynthesis was delayed in the RNAi-knockdown barley lines [22]. This and other studies have focussed on how WHY1 functions in the chloroplasts control organelle development and little attention has been paid to how WHY1 might regulate biogenic controls in the nuclei to stimulate chloroplast development. Our aim in the following studies was therefore to investigate the hypothesis that WHY1 regulates nuclear gene expression to trigger chloroplast development. To test this hypothesis, we have interrogated how the developmental patterns of transcripts and metabolites is changed in the developmental gradient in barley leaves. We firstly investigated the intracellular distribution of WHY1 between proplastids and nuclei in the bases of developing wild type barley leaves. We next characterised the developmental transcript and metabolite profiles that occur along barley lines and then determined how this pattern is changed in genotypes (W1–1 and W1–7) lacking WHY1. We discuss the evidence showing that the delay in plastid development observed in barley lines lacking WHY1 results from functions of the protein in the nuclei as well as the plastids. This research advances our understanding of WHY1 protein functions firstly by demonstrating that most of the WHY1 protein is localised in the nuclei at the early stages of leaf development, and secondly that this localisation is required for the appropriate expression of key nuclear genes such as GATA, GLK1-like and ARF transcriptions factors that influence chloroplast development.

## Results

### Expression and localisation of WHY1 in wild type and WHY knockdown barley leaves

**Figure 1.**
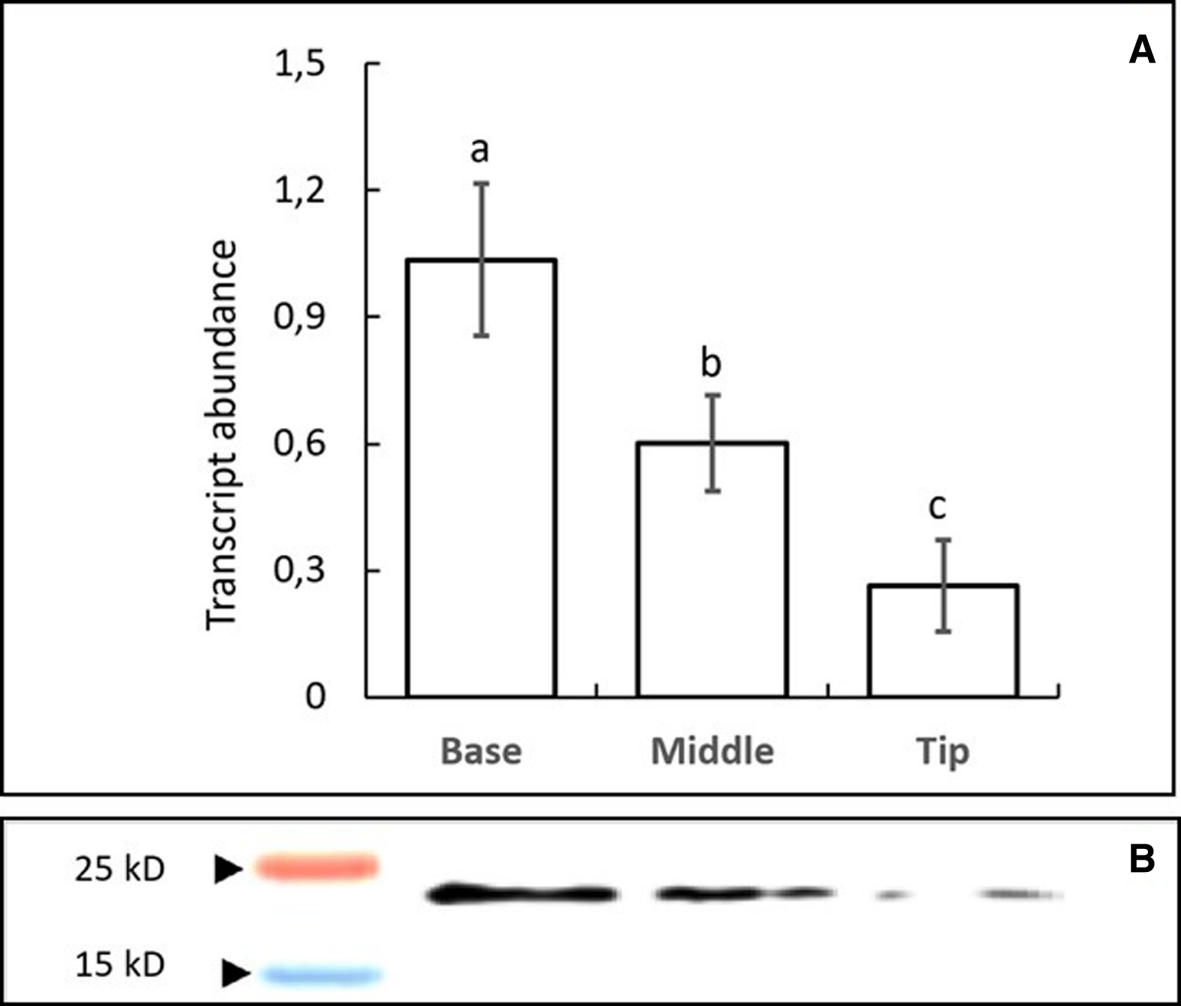
The relative abundance of WHY1 transcripts (A) and protein (B) in the base, middle and tip sections of the first leaf of 7-day old wild type seedlings estimated by qRT-PCR and Western blot, respectively. Data in panel A are presented as means ± SE (*n* = 3). Different letters represent statistical differences assessed by One-way ANOVA followed by Tukey's *post hoc* test (*P* < 0.05).

**Figure 2.**
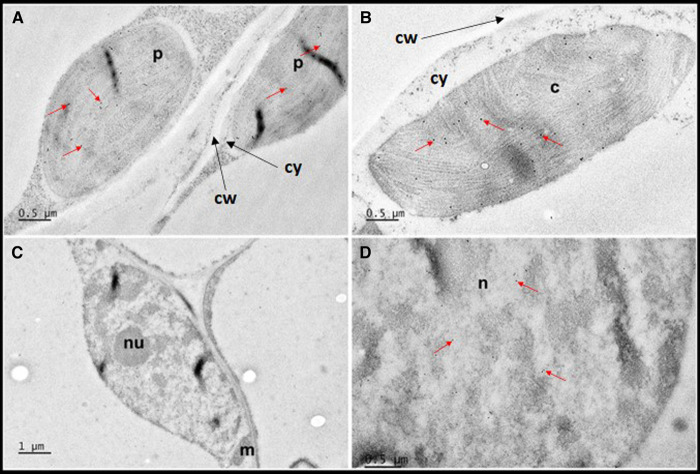
Distribution of WHY1 in barley leaf determined by immunogold labelling. Leaf sections were prepared for immunogold labelling and the presence of WHY1 detected by ployclonol antibody as described. Images are representative of plastids (p) in the leaf base (**A**); chloroplasts (c) in the leaf tip (**B**); nucleus with nucleolus (nu) in the leaf base (m, mitochondrion) (**C**) and a detail of the nucleus in the leaf base (**D**). In **A** and **B**, strips of cytoplasm (cy) can be observed as granular material between the organelles and the cell wall (cw). Example gold particles are indicated by red arrows. Scale bars representing 0.5 (**A**,**B**,**D**) or 1 µm (**C**) are provided in individual panels.

Since monocotyledonous leaves show a gradient of development from base to tip, we sampled the base and tip of the first leaves of 7-day old wild type seedlings to establish the intracellular localisation of WHY1. The levels of WHY1 transcripts were highest in the basal regions of 7-day old wild-type leaves decreasing progressively from the middle to tip (Figure 1). Immunogold labelling revealed that the WHY1 protein was found in developing plastids of the cells in the lowest region of the leaf bases that are not directly exposed to light [26] (Figure 2A), as well as in mature chloroplasts in the leaf tip (Figure 2B). Furthermore, imaging of the nucleus in the leaf base revealed that gold labelling was abundant (Figure 2C,D) where it appeared to be mostly clustered with electron dense chromatin (Supplementary Figure S1). In the leaf base we observed an average density of 15.88 ± 2.04 (*n* = 8) gold particles in plastids and 26.30 ± 2.40 (*n* = 10) gold particles in nuclei which was significantly greater (*P* < 0.005) as estimated using the student's *t*-test. Due to the presence of large vacuoles, we were unable to obtain high quality nuclear images from the leaf tip and hence were unable to measure the distribution of WHIRLY protein between the two compartments in older leaf sections. Labelling was largely absent from the cytosol (Figure 2).

WHY1-deficient barley leaves develop in a similar manner to the wild type except that the greening of each leaf is delayed in the absence of WHY1 [22]. To obtain an understanding of developmental delay we divided leaves into three sections; base, middle and tip. The basal sections of 7-day old wild type barley leaves had significantly less chlorophyll (Supplementary Figure S2) than the middle and tip sections. A similar chlorophyll gradient was observed in W1–1 and W1–7 leaves although the chlorophyll content in each section was lower than in the corresponding wild-type leaves (Supplementary Figure S2).

### Transcript profiles of leaf development indicate co-ordinated cell development and maturation

To obtain a broader understanding of developmental processes in wild-type plants we conducted a transcriptomic comparison of the base, middle and tip regions of seven day old leaves. One-way analysis of variance identified 440 transcripts that were significantly differentially abundant (*P* < 0.05) in the different leaf regions. An initial gene ontology (GO) enrichment analysis was conducted using the AgriGO tool [27]. This revealed significant enrichment in terms associated with plant growth and development such as ‘developmental processes', ‘anatomical structure development', ‘system development' and ‘post-embryonic development' (Supplementary Table S1). Moreover, terms associated with cell wall (e.g. ‘cell wall biogenesis’, ‘cell wall organisation', ‘lignin biosynthetic process') and lipid (e.g. ‘lipid biosynthetic process', ‘fatty acid biosynthetic process', ‘cellular lipid metabolic process') metabolism were prevalent in the list of significantly enriched GO terms (Supplementary Table S1).

**Figure 3.**
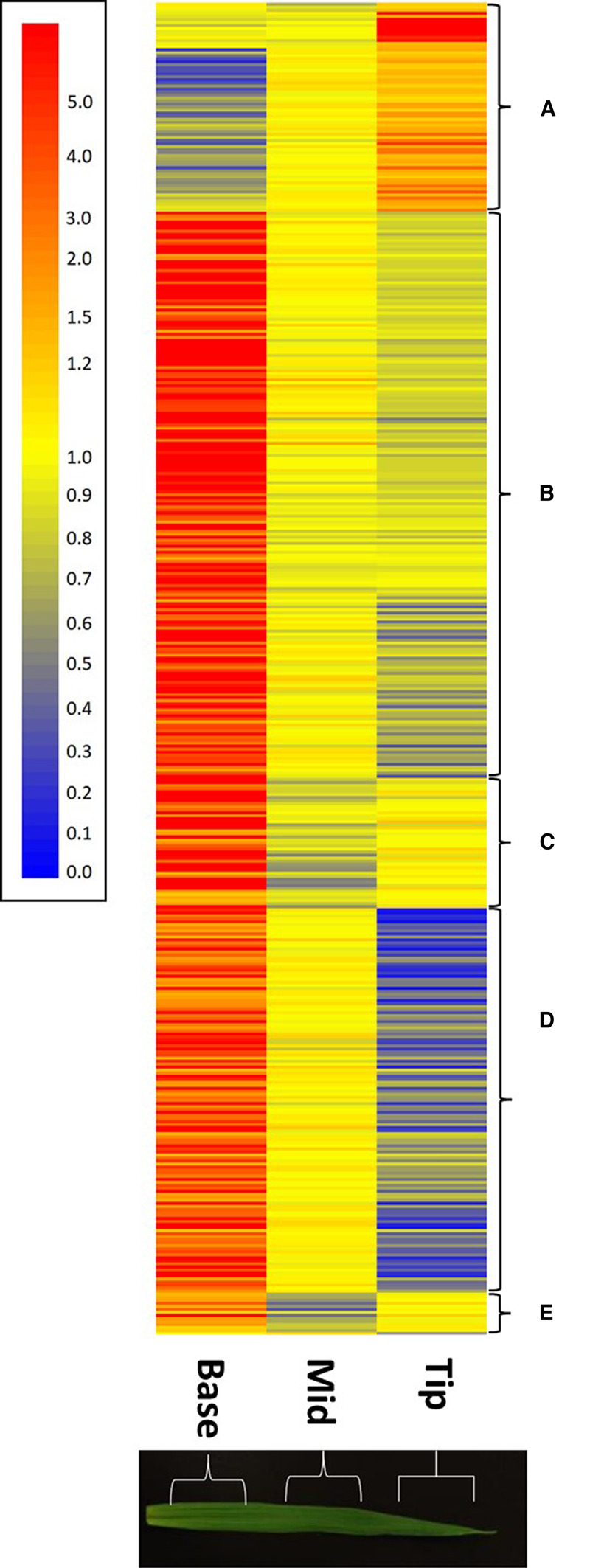
Cluster analysis comparison of abundance of transcripts that differ significantly between the base, middle and tip regions of wild type 7-day old barley leaves. Relative transcript abundance is represented according to the scale shown with highly abundant transcripts in red tones and less abundant transcripts in yellow and blue tones. Each transcript is represented by a single horizontal bar and transcripts were grouped in clusters (**A**–**E**) dependent on their patterns of abundance as indicated. The identity of individual transcripts are presented in order in Supplementary Table S1.

To gain a better understanding of the spatial and developmental expression of genes, transcripts were subjected to hierarchical clustering analysis to cluster those exhibiting similar developmental profiles. This analysis revealed five major clusters (Figure 3). In line with our GO enrichment analysis we focussed subsequent and our hypothesis that WHIRLY1 plays a role in leaf development and plastid biogenesis, we targeted our analysis to transcripts with functions in leaf and plastid biogenesis and development.

Cluster A comprised 69 transcripts that exhibited a gradient of abundance from low in the leaf base to high in the tip. This cluster included six transcripts encoding transcription factors homologous to Arabidopsis transcripts that have roles in leaf development (Supplementary Table S2). MLOC_74058.1 exhibits homology to an Arabidopsis transcription factor NGATHA3 (AT1G01030) involved in the control of leaf shape and expressed in leaf tips under the control of TCP (TEOSINTE BRANCHED 1, CYCLOIDEA and PROLIFERATING CELL FACTOR) transcription factors [28]. The latter family were represented by MLOC_14785.1 which exhibited homology to Arabidopsis TCP5 (AT5G60970). A gene (MLOC_70809.1) encoding a homologue of Arabidopsis GATA, NITRATE-INDUCIBLE, CARBON METABOLISM INVOLVED (GNC) transcription factor (AT5G56860) that regulates stomatal development, greening and chloroplast development [29–31] was also present in cluster A.

Two of the transcription factors identified within cluster A (Figure 3) were associated with the control of senescence in response to metabolic signals. AK373121 exhibits homology to an Arabidopsis zinc finger family protein METHYLENE BLUE SENSITIVITY 1 (MBS1; AT3G02790) responsible for acclimation or cell death in dose-dependent response to ^1^O_2_ [32]. MLOC_64240.2 and MLOC_53744.1 both share homology to AT1G56010 encoding NAC1, a senescence associated transcription factor under the control of auxin [33]. Further evidence for the up-regulation of senescence-associated processes in the leaf tip was the increased abundance of transcripts (AK370424, MLOC_47161.1) encoding proteins with homology to AUXIN-INDUCED IN ROOT CULTURES 3 (AT2G04160) and SENESCENCE-ASSOCIATED GENE 12 (AT5G45890; SAG12), endopeptidases required for protein turnover [34,35]. Furthermore, several transcripts (MLOC_56129.2, MLOC_57630.1, AK374126) encoding proteins homologous to proteins required for ubiquitin mediated protein turnover exhibited greatest abundance in the leaf tip (Supplementary Table S2).

Cluster B was the largest of the clusters comprising 187 transcripts that exhibited a gradient of abundance from high to low from the leaf base to the leaf tip (Figure 3). Seventeen transcripts encoding transcription factors were identified, several of which exhibited homology to Arabidopsis transcripts with functions in photomorphogenesis and development. Two transcripts (AK364144, MLOC_73144.4) showed homology to Arabidopsis auxin response factors (AT4G30080, AT1G19220; ARF) with functions in leaf morphogenesis and development [36,37]. Similarly, AK376150 and AK365841 are homologues of Arabidopsis genes encoding INDETRMINATE DOMAIN 15 (AT2G01940) and GATA TRANSCRIPTION FACTOR 2 (AT2G45050), with functions in leaf morphogenesis and photomorphogenesis, respectively [38,39]. As described below, a feature of cluster B were large numbers of transcripts associated with lipid and wax metabolism. Interestingly, we identified a transcript (AK364135) with homology to an Arabidopsis transcript encoding the class I TCP transcription factor TCP14 (AT3G47620). In Arabidopsis class I TCP transcription factors including TCP14 are master regulators of cuticle biosynthesis [40] and are required for the induction of genes involved in gibberellin biosynthesis and cell expansion in response to temperature [41]. Similarly, several transcripts in cluster B were associated with polyphenol metabolism and a transcription factor (AK361986) homologous to Arabidopsis MYB4 (AT4G38620) which functions in the control of flavonoid biosynthesis [42] was also identified in this cluster.

Consistent with previous observations that cells at the base of monocotyledonous leaves are undergoing division and expansion [43], 14 transcripts categorised as cell wall associated were identified in cluster B (Supplementary Table S2). These included several transcripts (AK248822.1, AK356936, MLOC_36439.1, MLOC_43237.1, MLOC_12096.1, MLOC_73204.3) with homology to transcripts encoding Arabidopsis expansins with a well-established role in cell wall loosening, leaf initiation and subsequent growth [44]. A further three transcripts (MLOC_61972.1, AK361522, AK361278) encoded xyloglucan endotransglycosylases (XTHs) that function in cell expansion by loosening cell walls [45]. Furthermore, transcripts encoding two pectin modifying enzymes, a methylesterase (MLOC_54267.1) and an acetylesterase (MLOC_55102.5) were highly abundant in the leaf base.

Transcripts associated with lipid metabolism were also highly represented within cluster B, consistent with previous observations that active cuticle biosynthesis is occurring in the basal portion of monocotyledonous leaves [46,47]. For example, MLOC_67622.1 and MLOC_45058.1 both exhibited homology to Arabidopsis transcripts encoding 3-KETOACYL-COA SYNTHASE 6 (KCS6, AT1G68530). Plants carrying mutations in *KCS6* exhibited significant reductions in branched and unbranched long chain alkanes and alcohols in cuticular wax [48]. Similarly, AK252678.1 shared homology with AT5G43760 encoding KCS20 a very long chain fatty acid synthase required for cuticular wax and root suberin biosynthesis [49]. Two transcripts (MLOC_54056.1 and AK370579) shared homology with AT1G02205 encoding ECERIFERUM 1, mutants of which exhibit similar defects to *KCS6* mutant lines [48]. Taken together these data suggest that the basal portion of the leaf comprises actively dividing/expanding cells.

Cluster C (Figure 3) comprised 43 transcripts that were abundant at the leaf base, scarce in the middle section of the leaf with intermediate abundance in the leaf tip. Cluster D comprised 127 transcripts that exhibited a similar high to low abundance profile during leaf maturation as observed for cluster B. However, the expression gradient in cluster D was considerably greater than observed for cluster B. Like cluster B, cluster D contained several transcripts encoding proteins associated with cell wall metabolism including expansins, XTHs and pectin modifying enzymes (Supplementary Table S2). Cluster D additionally contained transcripts encoding proteins required for cellulose biosynthesis where two transcripts (MLOC_66568.3, MLOC_68431.4) exhibited homology to Arabidopsis cellulose synthases (AT5G44030, AT5G17420) and a further two transcripts (MLOC_7722.1, AK370617) exhibited homology to an Arabidopsis transcript encoding the membrane anchored COBRA-LIKE 4 (AT5G15630) which plays a key function in cellulose deposition [50].

Furthermore, cluster D contained transcripts associated with cell expansion, cell polarity, organ patterning and development. MLOC_53132.1 exhibited homology to Arabidopsis transcripts (AT4G08950) encoding EXORDIUM, a brassinosteroid responsive gene that acts upstream of wall-associated kinases and expansins to promote cell expansion [51]. Indeed, a transcript (AK364262) with homology to the Arabidopsis transcript encoding WALL-ASSOCIATED KINASE 2 (AT1G21270) required for turgor driven cell expansion was also identified [52]. Several transcripts associated with vascular development and patterning were present. MLOC_58644.1 and AK359559 exhibited homology to Arabidopsis AT2G34710 and AT5G62880 encoding PHABULOSA and ROP11, respectively, which play roles in xylem patterning during early cellular differentiation [53,54]. Furthermore, MLOC_69397.2 homologous to an Arabidopsis transcript (AT1G79430) encoding ALTERED PHLOEM DEVLOPMENT with a role promoting phloem development was present [55]. Finally, a transcript encoding a basic-helix-loop-helix transcription factor (MLOC_55768.1) with similarity to an Arabidopsis transcript encoding bHLH93 (AT5G65640) was also present. This transcription factor interacts with FAMA which controls differentiation of guard cells in the leaf epidermis [56]. Taken together these results imply active cellular expansion and differentiation in the base of the leaf and indicate that these processes are complete in the more mature leaf regions. Transcripts in cluster E displayed a similar pattern of abundance to those in cluster C with minimum abundance in the mid region of the leaf.

### Loss of WHY1 has specific effects on leaf transcript abundance

Knockdown of the WHIRLY protein clearly delayed greening of emerging barley leaves (Supplementary Figure S2). To determine whether the WHIRLY protein influences other aspects of leaf development, a comparative transcriptome analysis of basal, mid and tip sections of leaves of wild type and two whirly knockdown lines was conducted. Significant differences in transcript abundance (*P* < 0.05) based upon leaf position, genotype or the interaction of the two factors was determined using two-way analysis of variance. This revealed 1732 transcripts that varied dependent on line, 2240 transcripts dependent on leaf region and only 23 which exhibited a distribution in abundance based on an interaction between the two factors (Supplementary Table S3).

Of the 23 transcripts exhibiting a genotype by leaf region interaction in their patterns of abundance, three transcripts (AK370975, MLOC_56051.1, MLOC_56052.1) that exhibited homology to chlorophyll binding proteins had a low abundance in all sections of wild-type leaves and although at higher abundance in W1–1 leaves both genotypes exhibited a pattern of reducing abundance from base to tip. In contrast, W1–7 leaves exhibited a reverse pattern of abundance increasing from base to tip. A similar pattern of abundance was observed for a transcript encoding a thylakoid luminal protein (AK370198). These data are consistent with delayed assembly of the photosystems in WHY-deficient leaves. Two other transcripts encoding proteins with functions in plastid biogenesis and metabolism also exhibited a genotype by leaf region dependent expression pattern. MLOC_9203.2 encodes a tubulin-like protein with homology to Arabidopsis AT2G36250 encoding an FtsZ protein essential for chloroplast division [57] while MLOC_69205.1 encodes an oxaloacetate/malate antiporter acting as a malate valve to balance NADPH/ATP ratios in the plastid [58].

**Figure 4.**
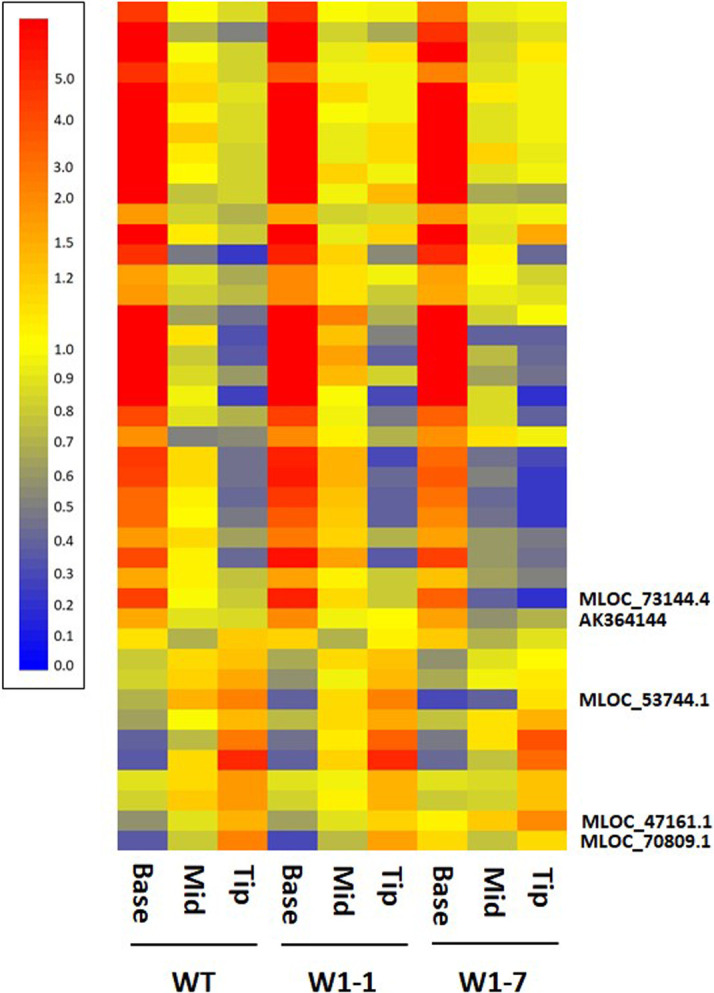
Cluster analysis comparison of selected transcripts in the base, middle and tip regions of wild-type, W1–1 and W1–7 barley genes. Transcripts were selected for analysis based upon their previously described association with leaf growth and development and their statistically significant differential abundance in different sections of wild-type leaves. Individual transcripts are represented by individual horizontal bars and relative transcript abundance is represented according to the legend shown where highly abundant transcripts are indicated in red and low abundance transcripts indicated in blue. The majority of transcripts exhibited similar developmental profiles of expression independent of plant genotype although a small number showed genotype dependent developmental profiles. Of the latter, key transcripts mentioned in the text are indicated to the right of the figure while the order of all transcripts represented is provided in Supplementary Table S3.

To understand how knock down of the WHIRLY protein influenced leaf development, we examined the abundance of key transcripts that exhibited changing developmental profiles in wild-type leaves discussed above in W1–1 and W1–7 leaves (Figure 4 and Supplementary Table S4). Of these transcripts, most exhibited similar patterns of abundance in the developmental profiles of all genotypes. Notable exceptions were MLOC_70809.1 the previously discussed GNC transcription factor (AT5G56860) that regulates stomatal development, greening and chloroplast development, MLOC_53744.1 encoding senescence-associated NAC1, SAG12 (MLOC_47161.1) and two transcripts encoding ARF transcriptions factors (AK364144, MLOC_73144.4) with functions in leaf morphogenesis and development. The patterns of abundance of these transcripts were perturbed in the absence of WHIRLY1 relative to wild type. While SAG12 transcripts were more abundant in all the sections of the W1–7 line than the other genotypes, the ARF and NAC1 transcripts were less abundant in all sections relative to the other genotypes (Figure 4). These data suggest that divergent developmental programmes observed between wild-type leaves and those deficient in the WHY1 protein are dependent on only a small number of transcripts.

### Transcript analysis of the basal section of leaves suggests delayed plastid development and perturbation of plastid gene expression in WHY1 knockdown lines

In wild-type leaves, transcripts associated with key developmental processes were highly abundant in the basal section of leaves and declined in the middle and tip sections. We therefore conducted one-way ANOVA to determine significantly differentially abundant transcripts in the base of wild type, W1–1 and W1–7 leaves. This identified 1267 transcripts that were differentially abundant dependent on genotype of which 540 were not assigned a function using the MapMan tool (Supplementary Table S5). The remaining 727 transcripts were assigned to a range of functions associated with primary and secondary metabolism, RNA, DNA and protein processing, cellular organisation and development (Supplementary Table S5).

**Figure 5.**
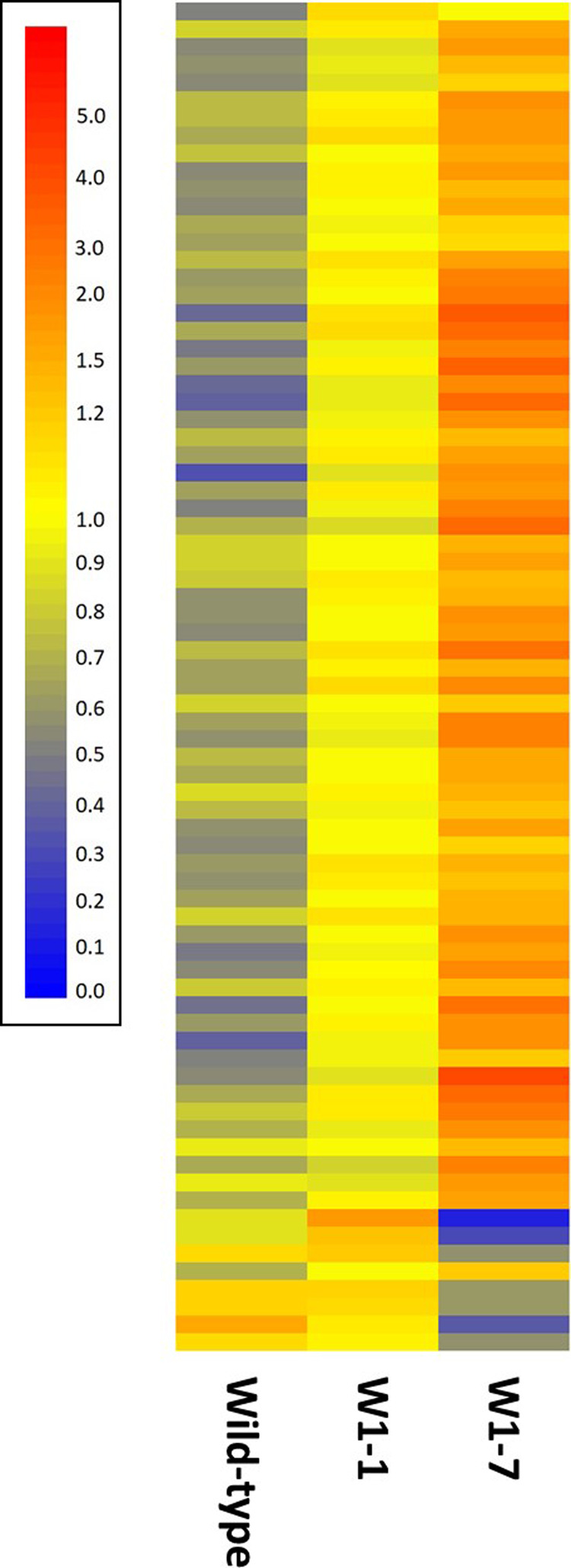
Cluster analysis comparison of abundance of transcripts with functions in plastid biogenesis and development that exhibit statistically significant differences in abundance in the base of wild type, W1–1 and W1–7 7-day old barley leaves. Relative transcript abundance is represented by the colour of horizontal bars according to the legend shown where red hues indicate highly abundant and blue hues less abundant transcripts. The identity of individual transcripts is provided in Supplementary Table S5 that list transcripts according to the order shown.

Seventy-six transcripts encoding proteins associated with plastid biogenesis and development were differentially expressed, (Figure 5 and Supplementary Table S6) the majority of which were more abundant in the base of 7-day old leaves of WHY1 knockdown plants than the wild type. These included transcripts encoding elements of the photosynthetic electron transport chains and Calvin cycle proteins including Rubisco (Supplementary Table S6). Furthermore, several transcripts (AK248405.1, MLOC_39198.3, MLOC_52167.2 and MLOC_63408.1) homologous to Arabidopsis transcripts encoding proteins required for FeS cluster assembly [59–62] were less abundant in the wild type than WHY1. Many transcripts encoded proteins associated with the targeting and translocation of proteins to the chloroplast as well as several proteins associated with protein folding. Examples include AK250352.1 and AK361117 both homologous to AT3G60370 encoding an immunophillin with protein folding activity required for the assembly of PSII [63]. Similarly, AK251420.1 encoding a chaperonin homologous to AT2G28000 required for plastid division [64] was less abundant in wild-type leaf bases relative to WHY1 leaf bases. Transcripts homologous to Arabidopsis transcripts encoding several plastid localised Clp proteases were also present including several (MLOC_32972.1, MLOC_4257.1, MLOC_64141.1, MLOC_68297.2, MLOC_861.2) that were previously demonstrated to play a role in plastid biogenesis [65].

A small number of transcripts exhibited a pattern of low abundance in W1–7. These included two transcripts (MLOC_56051.1, MLOC_56052.1) similar to AT2G34420 encoding a chlorophyll a–b binding protein and MLOC_61567.1 similar to plastid encoded ATCG01010 encoding an NADH dehydrogenase subunit. Interestingly, the levels of two transcripts (MLOC_65876.1, AK353571) with similarity to an Arabidopsis transcript (AT2G20570; GLK1) encoding a transcription factor required for the expression of nuclear encoded photosynthetic genes [66] were lower in the WHY1–7 leaves.

### Leaf developmental stage influences primary metabolite profiles

**Figure 6.**
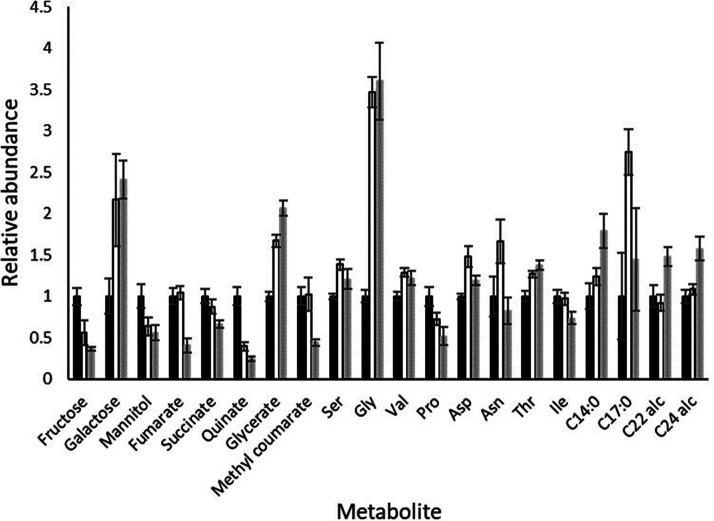
Polar and non-polar metabolites exhibiting significant differences in abundance in base, middle and tip regions of wild type 7-day old barley leaves. Abundance is shown relative to basal regions for each compound where bars represent mean and lines SE (*n* = 4). ▪, base; □, middle; ▪, tip. C14:0, tetradecanoic acid; C17:0, heptacanoic acid; C22 alc, docosanol; C24 alc, tetracosanol.

To identify shifts in metabolism associated with leaf development, untargeted GC/MS analysis of a range of polar and non-polar compounds was undertaken. A total of 107 chromatographic features were resolved representing 36 non-polar and 71 polar components. Thirty of the features were unidentified with the remaining 77 identified based on their relative retention times and mass spectra. Only 26 components exhibited significant differences in abundance dependent on leaf section of which 19 were present in the polar fraction and 7 in the non-polar fraction (Supplementary Table S7). Twenty of these compounds were identified comprising sugars, organic acids, amino acids, fatty acids and alcohols (Figure 6). A minor unoximated fructose peak exhibited a decline from leaf base to tip however, this represented less than 10% of the total fructose pool and neither of the major oximated peaks exhibited significant change (Supplementary Table S7). Mannitol exhibited a similar change in abundance to the unoximated fructose peak while galactose was least abundant in the leaf base but higher in the mid and tip regions (Figure 6). The TCA cycle organic acids fumarate and succinate were most highly abundant in the base and mid regions of the leaf but declined dramatically in the leaf tip perhaps due to a redirection of flux into amino acids, the majority of which exhibited an increase as the leaf aged (Figure 6). Significantly, although both glycine and serine increased as the leaf aged, glycine increased to a much greater extent meaning a higher glycine/serine ratio indicative of increased photorespiration [67] from leaf base to tip. Of the four lipophilic compounds that exhibited significant changes in abundance, all increased in the leaf tip relative to the leaf basal region.

### Loss of WHY1 has specific effects on C/N metabolism

**Figure 7.**
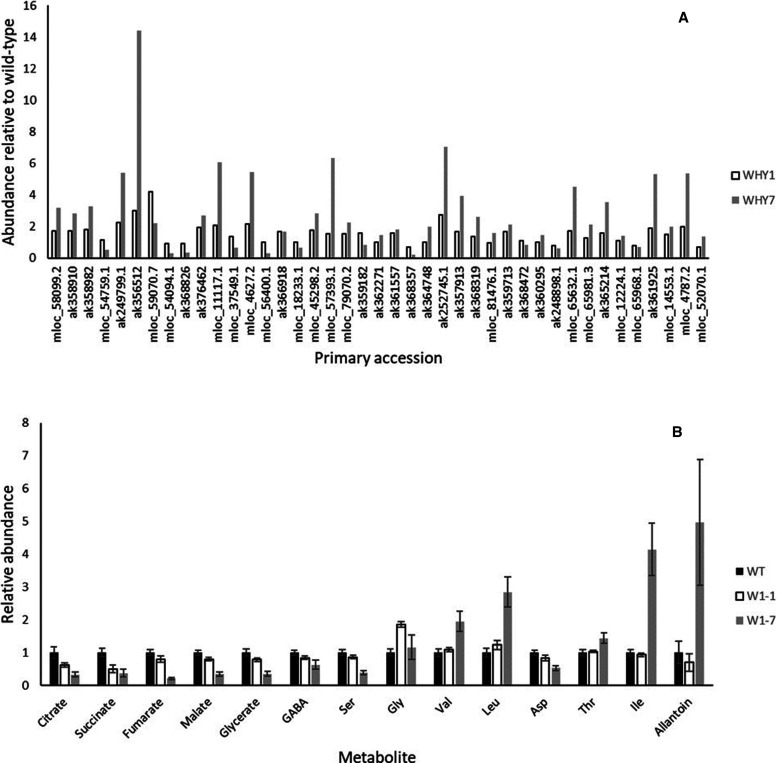
Relative abundance of transcripts associated with primary metabolism (A) and primary metabolites (B) exhibiting significant differences in abundance in the basal region of 7-day old wild type and WHY1 knockdown barley leaves. Transcript annotations are provided in Supplementary Table S7.

Several transcripts associated with primary metabolic pathways were differentially abundant in the basal portion of wild type and WHY1 knockdown leaves (Figure 7A). Significant differences were observed in transcripts encoding enzymes associated with the Calvin cycle, starch and sugar metabolism, glycolysis, the TCA cycle and amino acid metabolism (Supplementary Table S8). Many of these transcripts were more abundant in WHY1 knockdown than wild-type leaves. However, transcripts encoding hexokinase (MLOC_54094.1), β-amylase (AK368826) a methionine S-methyltransferase (AK368357), an O-acetylcysteine thiol-lyase (AK248898.1) and an arginase (MLOC_65968.1) were consistently less abundant in WHY1 knockdown lines.

Differences in transcript abundance were reflected by significant differences in primary metabolic profiles (Figure 7B). Twenty-two of 71 polar compounds analysed by GC/MS were significantly differentially abundant in wild type and WHY1 knockdown leaf basal regions. Eight of these compounds were unidentified with the remainder comprising primarily organic and amino acids. All of the TCA cycle components detected were significantly lower in WHY1 knockdown leaves than in wild-type leaves as was the non-proteinaceous amino acid γ-amino butyric acid (GABA) that functions as a cytosolic bypass of specific steps [68]. Similarly, serine and aspartate were present at lower concentrations in WHY1 knockdown leaves while glycine, valine, leucine, threonine and isoleucine were present at higher concentrations, particularly in W1–7 (Figure 7B).

### Delayed accumulation of photosynthetic proteins in the WHY knockdown lines

**Figure 8.**
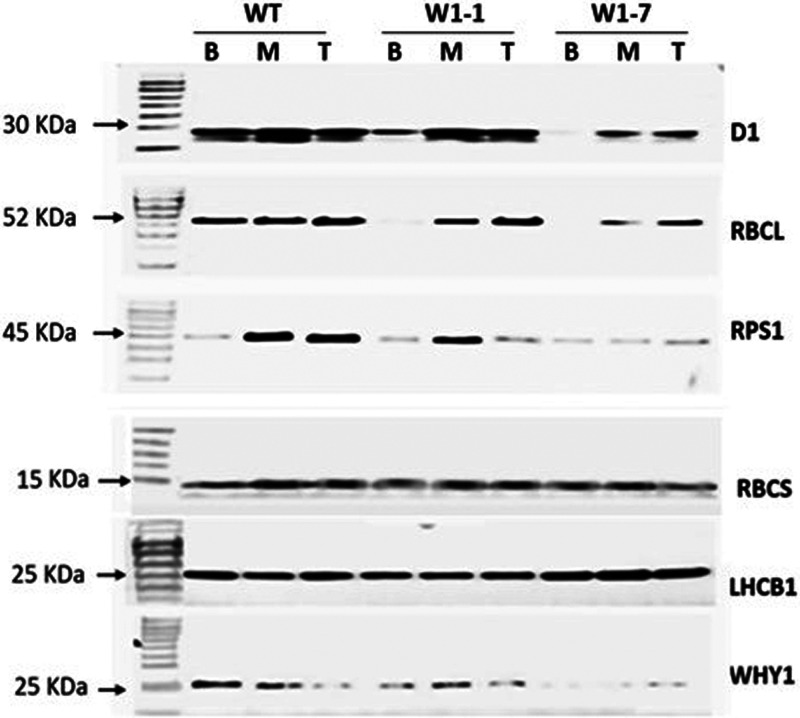
Western blots of selected chloroplast proteins in the base (B), middle (M) and tip (T) sections of the first leaves of wild-type (WT), W1–1 and W1–7 seedlings 7 days after germination. Proteins encoded by the plastid genome were detected as described and are chlorophyll a/b-binding proteins: LHCB1 and LHCB2, the small subunit of RUBISCO (RBCS), chloroplast ribosomal protein S1 (RPS1), the large subunit of RUBISCO (RBCL), the photosystem II protein (D1) and WHIRLY1 (WHY1). Molecular weight markers for each gel are indicated in lane 1 marked MW.

The levels of LHCBII protein were slightly higher in all regions of the leaves of 7-day old W1–1 and W1–7 seedlings than the wild type (Figure 8). In contrast, the D1 protein, the RPS1 and the RBCS and RBCL proteins were much less abundant in the leaves of 7-day old W1–1 and W1–7 seedlings than the wild type (Figure 8).

## Discussion

The concept that the distribution of WHY1 between the nuclei and chloroplasts plays a key role in the regulation of plant development has arisen in recent years [69,70]. The phosphorylation of the WHY1 protein favours partitioning to nuclei, a process that increases with leaf age [71]. However, little information is available on the distribution of WHY1 between the nuclei and proplastids of developing leaves. The immunogold labelling analyses reported here revealed that over 60% of the WHY1 protein in the basal sections of the leaves was located in the nuclei (Figure 2). Hence, it is likely that WHY1 fulfils its functions in the nuclei of developing leaves as well as the plastids. Chloroplast development is delayed in the absence of WHY1, a process that has largely been ascribed to the functions of WHY1 in chloroplasts [22,25]. However, WHY1 functions as a transcriptional activator in the nucleus, binding to the AT-rich region of the kinesin gene promoter to activate kinesin gene expression [72], and to the GTCAAT motif of the *S40* promoter [73], and to a combination motif of GTNNNAAT and AT-rich motif of downstream target genes, such as *WRKY53*, *WRKY33*, *SPO11*, and *PR1* to regulate leaf senescence and other processes in *A. thaliana* [69,74,75].

The above findings show that the developmental profile of the barley leaves was modified in the WHY1 plants. The changes provide the following insights into how WHY1 specifically influences the developmental profile of transcripts. WHY1 modulates the GNC transcription factor regulating stomatal development, greening and chloroplast development. It also influences NAC1, an auxin-regulated senescence-associated transcription factor, and two transcripts encoding ARF transcriptions factors with functions in leaf morphogenesis and development. Similarly, WHY1 regulates the abundance of two transcripts with similarity to Arabidopsis GLK1 that encodes a transcription factor required for the expression of nuclear-encoded photosynthetic genes [66]. WHY1 has been shown to regulate the expression of senescence associated genes in the nucleus [73]. Here we show that the absence of WHY1 led to higher levels of SAG12 transcripts in all the sections of the W1–7 line than the other genotypes, confirming the role of nuclear WHY1 in the control of barley leaf development. We conclude that the above changes in nuclear-encoded proteins that control chloroplast development are the targets of the WHY1 protein in the nucleus.

The WHY1 protein binds to ERF-binding *cis* elements in the promoter regions of genes such as ERF109 (REDOX RESPONSIVE TRANSCRIPTION FACTOR 1, RRTF1) [75]. ERF109 is involved in plant stress responses and participates in reactive oxygen species (ROS) signalling and the regulation of developmental programs, such as jasmonate-dependent initiation of lateral root development [76]. WHY proteins have previously been reported to be involved in the regulation of shoot and root development. For example, WHY2 was shown to be a major regulator of root apical meristem development [77]. Similarly, the expression of WHY1 (nWHY1) in the nucleus of Arabidopsis *why1* mutants led to changes in the levels of transcripts associated with plant development during early growth, whereas expression of WHY1 in plastids increased the abundance of transcripts associated with salicylic acid synthesis [78]. The binding of WHY proteins to the PB element of the *9-cis epoxycarotenoid dioxygenase (NCED)1* gene in cassava activated expression leading to increased abscisic acid levels [79]. Hence, the presence of WHY proteins in the nucleus clearly influences the expression of genes involved in the synthesis of phytohormones that control plant growth and defence. The primary cause of the delay in greening observed in the barley leaves lacking WHY1 may therefore result from the absence of WHY1-dependent regulation of nuclear gene expression.

The action of WHY1 as a transcription factor in the nucleus also regulates the expression of genes associated with photosynthesis and carbon metabolism. For example, WHY1 binds to the promoter of the rbcS gene that encodes the small subunit of the potato ribulose-1, 5-carboxylase, oxygenase under cold stress [80], while WHY2 binds to the promoters of the *SWEET11/15* genes that encode sucrose transporters, leading to the modulation of starch allocation and silique development [17]. Here we report that the absence of WHY1 has a significant impact on the levels of transcripts encoding enzymes associated with the Calvin cycle, starch and sugar metabolism, glycolysis, the TCA cycle and amino acid metabolism, many of which were more abundant in WHY1-deficient leaves than the wild type. However, transcripts encoding enzymes such as β-amylase were less abundant in WHY1 knockdown lines. WHY1 is known to bind to the ERE-like element of the *AMY3-L* promoter, activating the expression of amylase and starch degradation. WHY1 also binds to the ERE element of the *ISA2* promoter to inhibit isoamylase-mediated starch-synthesis [81]. The absence of WHY1 from the nuclei of developing barley leaves could therefore lead to the observed changes in primary metabolites reported here. For example, all the metabolites of the TCA cycle that were detected were significantly lower in WHY1 knockdown leaves than the wild type, as were GABA. Other amino acids such as glycine, valine, leucine, threonine and isoleucine were higher in WHY1-deficient leaves than the wild type. It may be that WHY1 can bind to promotors of a wide range of housekeeping genes in the nucleus, to modulate their expression in response to developmental and environmental signals.

WHY1 deficiency in barley leaves delays greening and the establishment of photosynthesis [22]. The absence of WHY1 in the developing chloroplasts prevents ribosome formation and the associated acquisition of photosynthetic activity [22]. The data presented here suggests that WHY1 functions in the nucleus are also important in the regulation of chloroplast development. Given the distribution of WHY1 in the basal regions of the developing leaves, in which 60% of the WHY1 protein is present in the nuclei and 40% in the proplastids, in addition to the effects of WHY1 deficiency on nuclear-encoded transcripts that are involved in chloroplast development, we propose that WHY1 participates in biogenic signalling and that it is required for co-ordinate control of nuclear and plastome gene expression that ensures accurate chloroplast biogenesis. There is other evidence that supports this concept. For example, plastid-localised WHY1 affects miRNA biogenesis in the nucleus [82].

The pioneering studies of Isemer et al. [83] demonstrated that while the sensitivity of germination in Arabidopsis seeds to ABA was dependent on whether WHY1 was located in the plastids or nuclei. When WHY1 was present in both compartments the seeds showed enhanced sensitivity to ABA. In contrast, seeds in which WHY1 was targeted only to the nuclei were insensitive to ABA as where the *why1* mutants. Such studies demonstrate that the intracellular localisation of WHY1 has a key role in the regulation of early plant development. A similar approach could be used to explore how the compartmentation of WHY1 in the proplastids and nuclei regulates biogenic anterograde and retrograde signalling between the nucleus and developing chloroplasts. The role of posttranslational controls in determining the intracellular localisation of WHY1 in such systems could be explored using appropriate mutants and transgenic lines. It is known that protein phosphorylation alters the partitioning of WHY1 between the nuclei and chloroplasts at the onset of senescence [69]. Phosphorylation of the WHY1 protein by CIPK14 kinase or oxidation caused a re-distribution of WHY1 from the plastids to the nuclei [69,70]. However, this may be only one of several mechanisms that facilitate the relocation of WHY1 between different intracellular compartments. Other evidence has shown that WHY1 can move from the plastids to the nucleus [84]. Direct transfer of WHY1 from the plastids to the nuclei through contact sites or stromules [85] is possible. Further exploration of the mechanisms underpinning the compartment-shifting of proteins, as discussed by Foyer et al. [86], will shed new light on how WHY1 controls early chloroplast development as well as senescence [69].

In conclusion, the studies reported here were undertaken to investigate whether WHY1 regulates nuclear gene expression in developing leaves in a manner that might regulate chloroplast development. Firstly, we interrogated the developmental patterns of transcripts and metabolites in the emerging leaves of barley seedlings. We have provided evidence that this pattern is changed in the absence of the WHY1 protein, as situation that leads to a delay in greening by ∼10 days. We show that WHY1 is predominantly localised in the nuclei of the leaf bases and that the expression of nuclear genes such as GATA, GLK1-like and ARF transcriptions factors that influence chloroplast development is changed when WHY1 is absent.

This study provides evidence that WHY1 contributes to the regulation of nuclear-encoded proteins that regulate chloroplast development.

## Materials and methods

### Plant material and growth conditions

Seeds of two independent transgenic barley (*Hordeum vulgare* L. cv. Golden Promise) lines (W1–1 and W1–7) with RNAi knockdown of the *WHIRLY1* gene and wild-type controls were produced as described previously [87]. Barley seeds (1 per pot) were sown in pots in compost (All Purpose Growing Medium, Sinclair, Cheshire, U.K.) in controlled environment chambers with a 16 h light/8 h dark photoperiod, with an irradiance of 250 µmol m^−2^ s^−1^, 20°C/16°C day/night temperature regime and 60% relative humidity. The primary leaves were harvested after 7 days.

### Leaf pigments

Leaf pigments were extracted from barley leaf sections, ground in liquid nitrogen and extracted with 80% (v/v) acetone. Absorbance of chlorophylls was measured at 663 and 646 nm and concentrations were calculated using the formula given by Lichtenthaler [88].

### qPCR

Reverse transcription of 1 µg of RNA into cDNA was performed using the QuantiTect Reverse Transcription Kit (Qiagen U.K., Manchester, U.K.). The qPCR was performed using QuantiFast SYBR Green PCR kit (Qiagen) in the presence of 0.5 µM primers in a CFX96 thermocycler (Bio-Rad, Hercules, CA, U.S.A.) following the manufacturer's instructions. PCR conditions were as follows: incubation at 95°C for 5 min, 45 cycles 10 s 95°C and 30 s 60°C. Additionally melting curve analysis was performed at the end of each run to ensure specificity of the products. The same master mix without cDNA was used as negative control. The following primers were used: WHY1 (AK365452.1) Fwd 5′-GATGGGAATGGTCGCTTTTT-3′, Rev 5′-CCATGATGTGCGGTATGATG-3′), ACTIN 11 (AY145451) Fwd 5′-CGACAATGGAACCGGAATG-3′, Rev 5′-CCCTTGGCGCATCATCTC-3′) and Elongation factor 1- a (Z50789) Fwd 5′-TTGGTGGCATTGGAACTGTG-3′Rev 5′-CAAACCCACGCTTGAGATCC-3′.

### Microarray processing and analysis

Microarray processing and analysis was performed on leaf RNA extracts from three biological replicates per treatment using a custom designed barley Agilent microarray (A-MEXP-2357; www.ebi.ac.uk/array-express) as previously described [89]. Raw data can be accessed via the array express website (www.ebi.ac.uk/array-express) using accession number (E-MTAB-9882).

### Metabolite profiling by GC/MS

GC/MS analysis was performed on extracts from four biological replicates per treatment. Leaf sections (base, mid, tip) were combined from three individual barley plants were combined, snap frozen in liquid N_2_ and lyophilised. An amount of 100 ± 5 mg of dried material was weighed into glass tubes and material was sequentially extracted in methanol, water and chloroform for 30 min each at 37°C as previously described [89]. Internal standards (ribitol and nonadecanoic acid) were added following the initial methanol addition. Finally, an additional aliquot of water was added, and the polar and non-polar phases were separated and converted to trimethylsilyl or methyl ester derivatives as previously described [90]. Metabolite profiles for the polar and non-polar fractions were acquired following separation of compounds on a DB5-MSTM column (15 m × 0.25 mm × 0.25 µm; J&W, Folsom, CA, U.S.A.) using a Thermo-Finnigan DSQ II GC/MS system. Data was then processed using Xcalibur software. Peak areas relative to internal standard were calculated following normalisation to 100 mg extracted material.

### Western blots

Total proteins were extracted with protein extraction buffer (Agrisera, Vannas, Sweeden) supplemented with 5 mM DTT and the cocktail of protease inhibitors to prevent protein degradation. An amount of 10 µg of proteins were separated on 15% acrylamide SDS–PAGE and transferred to 0.45 µm nitrocellulose membrane (Amersham, Buckinghamshire, U.K., catalogue number 10600003). All proteins apart from WHY1 were detected with rabbit polyclonal primary antibody (Agrisera) and secondary HRP-linked anti-rabbit (1 : 10000, Agrisera catalogue number AS09 602).

For immunological detection of WHY1, the antibodies were directed toward the synthetic peptide of recombinant HvWhy1 protein (PRQYDWARKQVF) in rabbits and antibodies were affinity-purified (Generon, Slough, U.K.). The specificity of immunodetection was validated using pre-immune sera.

### Immunogold labelling

Young barley leaves were cut transversely with a fresh razorblade into 1 mm diameter strips. The samples were then prepared for immunogold labelling (IGL) and transmission electron microscopy (TEM) according to Rubio et al. [91]. Briefly, they were fixed immediately in 4% paraformaldehyde + 0.5% glutaraldehyde in 0.05 M sodium cacodylate (pH 7.0) overnight. After dehydration in ethanol the samples were infiltrated and embedded in LR White resin. The leaf samples were sectioned on a Leica UCT ultramicrotome and the sections (80 nm) collected on Ni grids coated with pyroxylin. After 1 h blocking in IGL buffer [91] the grids were incubated for 2 h at room temperature in a polyclonal antibody (diluted 1 : 10 in IGL buffer) raised against barley WHIRLY1 in goat (Agrisera, Vannas, Sweden). After two washes in IGL buffer (10 min per wash) the grids were then incubated for 2 h in rabbit anti-goat IgG 10 nm gold (Aurion Immuno Gold Reagents & Accessories, Wageningen, Netherlands). The grids were finally washed twice in IGL buffer (5 min per wash) and 10 times in dH2O (30 s per wash). The immunogold labelled grids were viewed and photographed under a JEOL JEM1400 transmission electron microscope, and gold particles were counted on 8–10 representative images from each sample taken at the same magnification (×4000).

### Statistical analyses

Microarray data was analysed using GeneSpring (v. 7.3; Agilent) software. Data were normalised using default single-channel settings: intensity values were set to a minimum of 0.01, data from each array were normalised to the 50th percentile of all measurements on the array, and the signal from each probe was subsequently normalised to the median of its value across all samples. Unreliable data ﬂagged as absent from all replicate samples by the FE software were discarded. Statistical ﬁltering of data was performed using one- or two-way analysis of variance (ANOVA; *P* ≤ 0.05) for the factors ‘leaf region' and ‘genotype' with Bonferroni multiple testing correction.

Statistical analysis was carried out on the metabolite data sets collated from GC/MS polar and non-polar fractions. Firstly, principal components analysis (PCA), using the sample correlation matrix which gives equal weight to all metabolites, was used to summarise broad scale variation among the samples. A second approach involved an ANOVA of each individual metabolite using the main factors leaf region, genotype or an interaction of the two factors. Statistical analyses were performed using GenStat for Windows, 18th Edition (VSN International Ltd., Hemel Hempstead, U.K.).

## Data Availability

All primary data and datasets are available from the authors upon request.

## Open Access

Open access for this article was enabled by the participation of University of Birmingham in an all-inclusive *Read & Publish* agreement with Portland Press and the Biochemical Society under a transformative agreement with UK.

## Abbreviations

LHC: light harvesting chlorophyll a/b binding complex
MDS: mitochondrial dysfunction stimulon
n: nuclear
NEP: nuclear-encoded RNA polymerase
PEP: plastid-encoded RNA polymerase
*PhANGs*: photosynthesis-associated nuclear genes
pt: plastid
RBCL: large subunit of RuBiSCO
RBCS: small RuBiSCO subunit of
RBPs: RNA-binding proteins
RCD1: RADICAL-INDUCED CELL DEATH1
RuBiSCO: ribulose-1, 5-bisphoshate carboxylase
