# Supplementary material for: WHIRLY1 functions in the nucleus to regulate barley leaf development and associated metabolite profiles
Source: Biochem J. 2022 Mar 4;479(5):641–59. doi: 10.1042/BCJ20210810 (PMC9022988; doi:10.1042/BCJ20210810)
Supplement: Supplementary Material [file BCJ-479-641-s1.pdf]

Supplementary Figures and Tables

Figure S1: Representative transmission electron micrograph of nucleus in base of WT barley leaves indicating the association of WHY1 protein (red arrows) with electron dense chromatin (Ch).

Figure S2: A comparison of leaf chlorophyll content in the base, middle and tip sections of the first leaves of 7-day old wild type (WT), W1-1 and W1-7 seedlings. Data are presented as mean ± SE (n = 6). Different letters represent statistical differences assessed by one-way ANOVA followed by Tukey's *post hoc* test.

Table S1: Gene ontology (GO) term enrichment amongst 440 transcripts significantly differentially abundant dependent on sampling position (base, mid, tip) in wild-type barley leaves. Significantly ($P < 0.05$) enriched GO terms associated with biological processes were identified using the AgriGO tool (Tian et al., 2017) and are representation by their annotation number and description. The table additionally reports the number of gene annotations associated with each category and those present in the reference list. In this analysis, the nearest Arabidopsis homologue of the barley transcript was used to test for enrichment against the TAIR 10 Arabidopsis genome.

Table S2: Annotated transcripts in the base, middle and tip of 7-day old wild type leaves that exhibit significant changes in abundance dependent on leaf position. Transcripts are ordered as indicated in fig. 3.

Table S3: Transcripts in the base, middle and tip of 7-day old wild type, W1-1 and W1-7 barley leaves that exhibit significant changes in abundance dependent on genotype, leaf position or interaction of the two factors.

Table S4: Transcripts associated with cell wall metabolism that exhibit differential abundance in the base, middle and tip of 7-day old wild type barley leaves.

Table S5: Transcripts significantly differentially abundant in the basal region of wild type, W1-1 and W1-7 barley leaves.

Table S6: Transcripts associated with plastid biogenesis and development significantly differentially abundant in the basal region of wild type, W1-1 and W1-7 barley leaves. Transcripts are ordered in accordance with Fig. 5.

Table S7: Metabolites significantly differentially abundant in the base, middle and tip of 7-day old wild type barley leaves. Relative abundance is described as mean and standard error. P values represent significance and FDR represents the calculated rate of false positive. Unknown compounds identified in the non-polar (NP) or polar (P) fractions of leaf extracts are labelled according to the MS scan number of their peak apex, fatty acids are labelled according the convention Cx:y where x is the number of carbon atoms and y is the number of unsaturated bonds, fatty alcohols are labelled Cx alc where c is the number of carbon atoms, amino acids are labelled according to standard three letter abbreviations.

Table S8: Relative abundance of transcripts associated with carbon and nitrogen metabolism significantly differentially abundant in the basal region of wild type, W1-1 and W1-7 barley leaves.

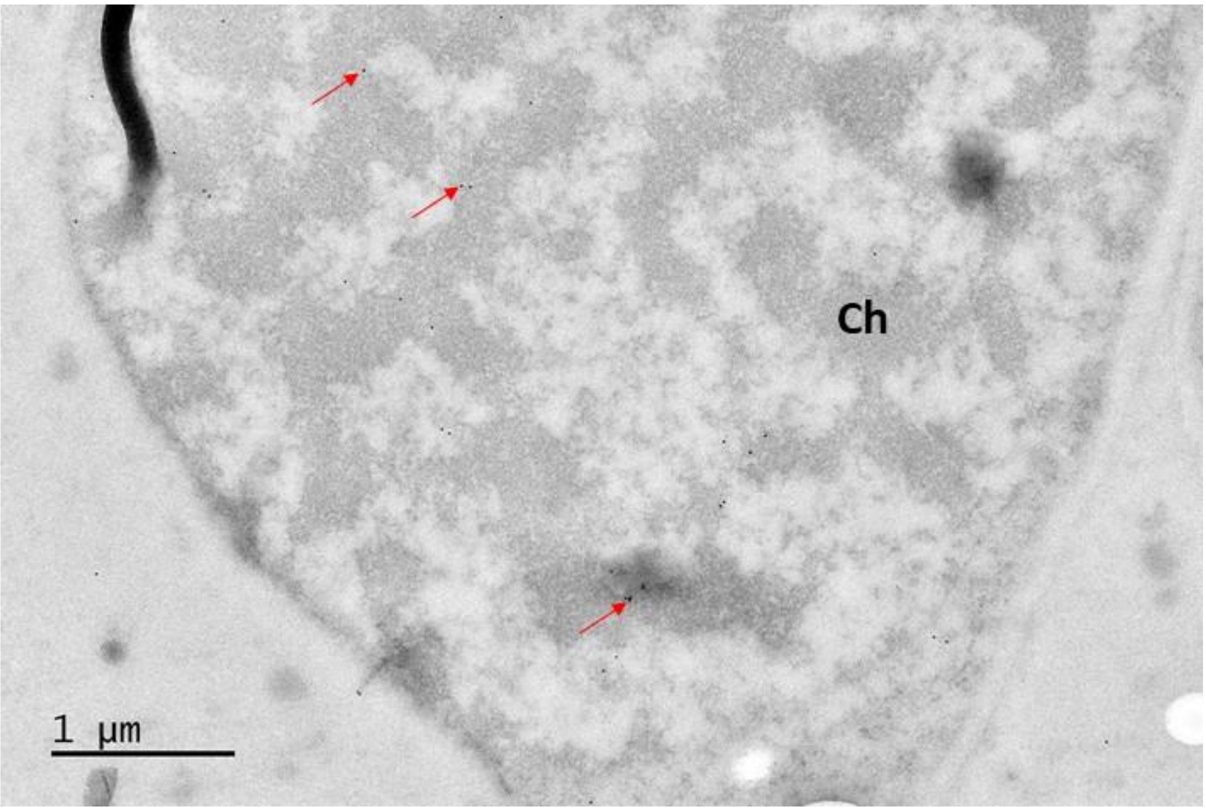
Ch
1 µm

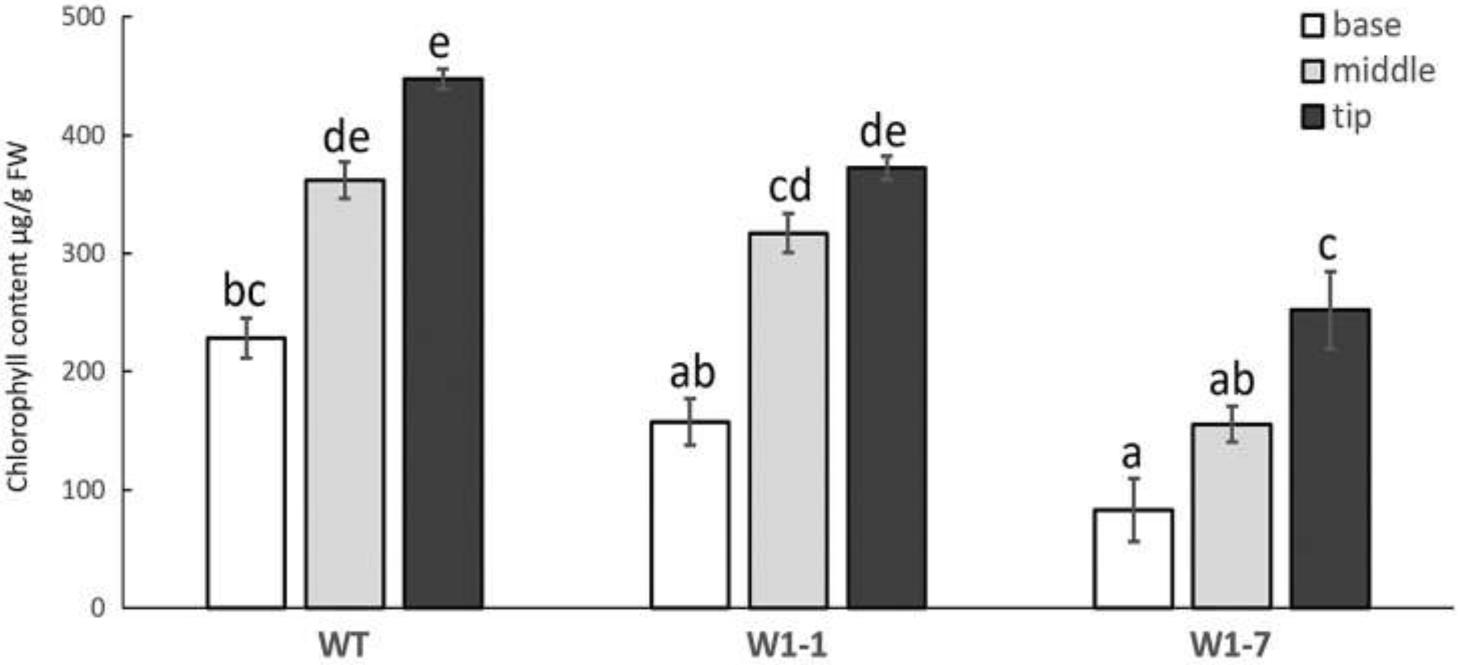

Chlorophyll content µg/g FW
500
400
300
200
100
0
base
middle
tip
e
de
bc
de
cd
ab
c
ab
a
WT
W1-1
W1-7
